# Supplementary figures and images for: Dissection of affinity captured LINE-1 macromolecular complexes
Source: eLife. 2018 Jan 8;7:e30094. doi: 10.7554/eLife.30094 (PMC5821459; doi:10.7554/eLife.30094)

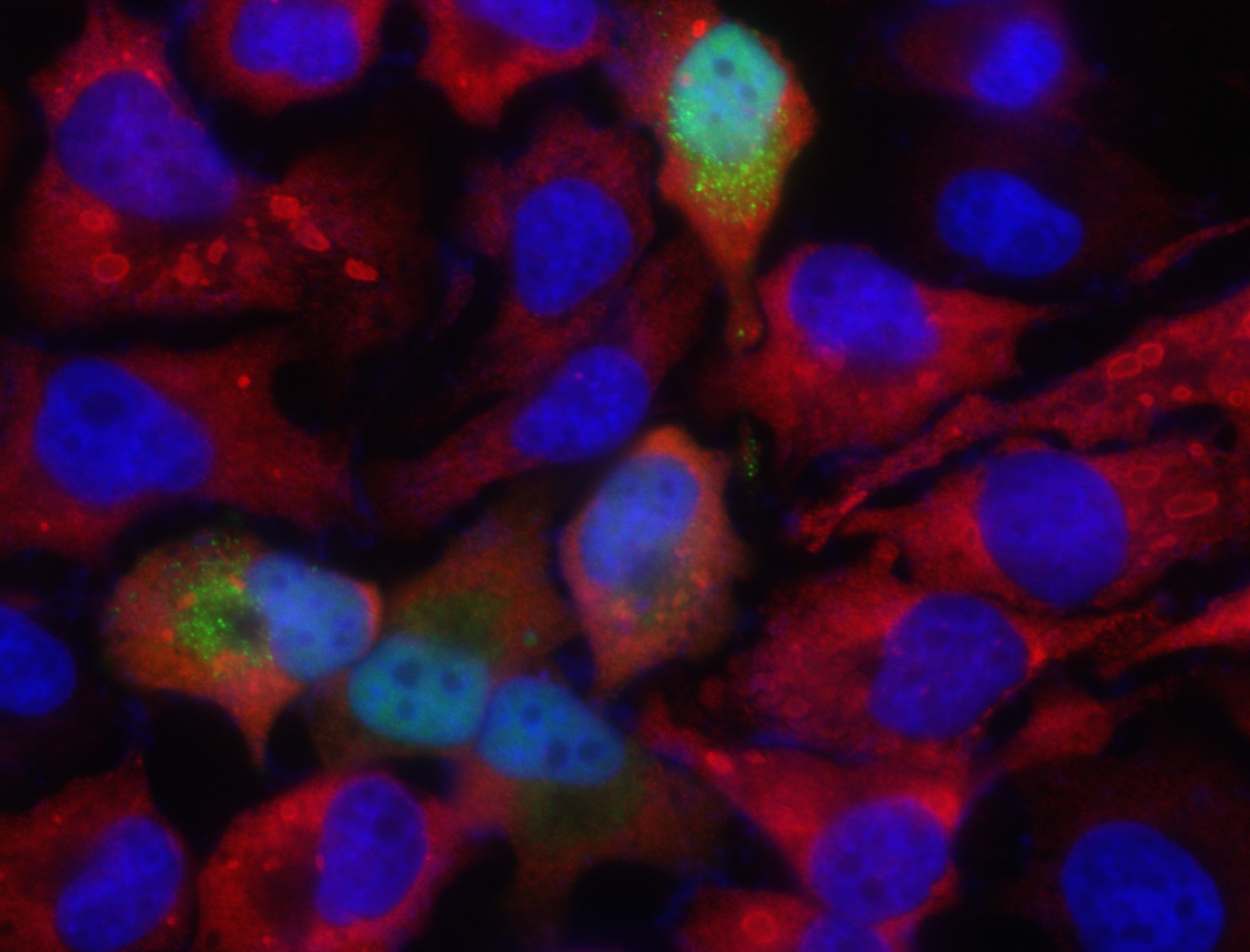

Supplement: Figure 3—source data 1. [file elife-30094-fig3-data1.zip › A_LD401 Fibro_001&-24Bit.jpg]

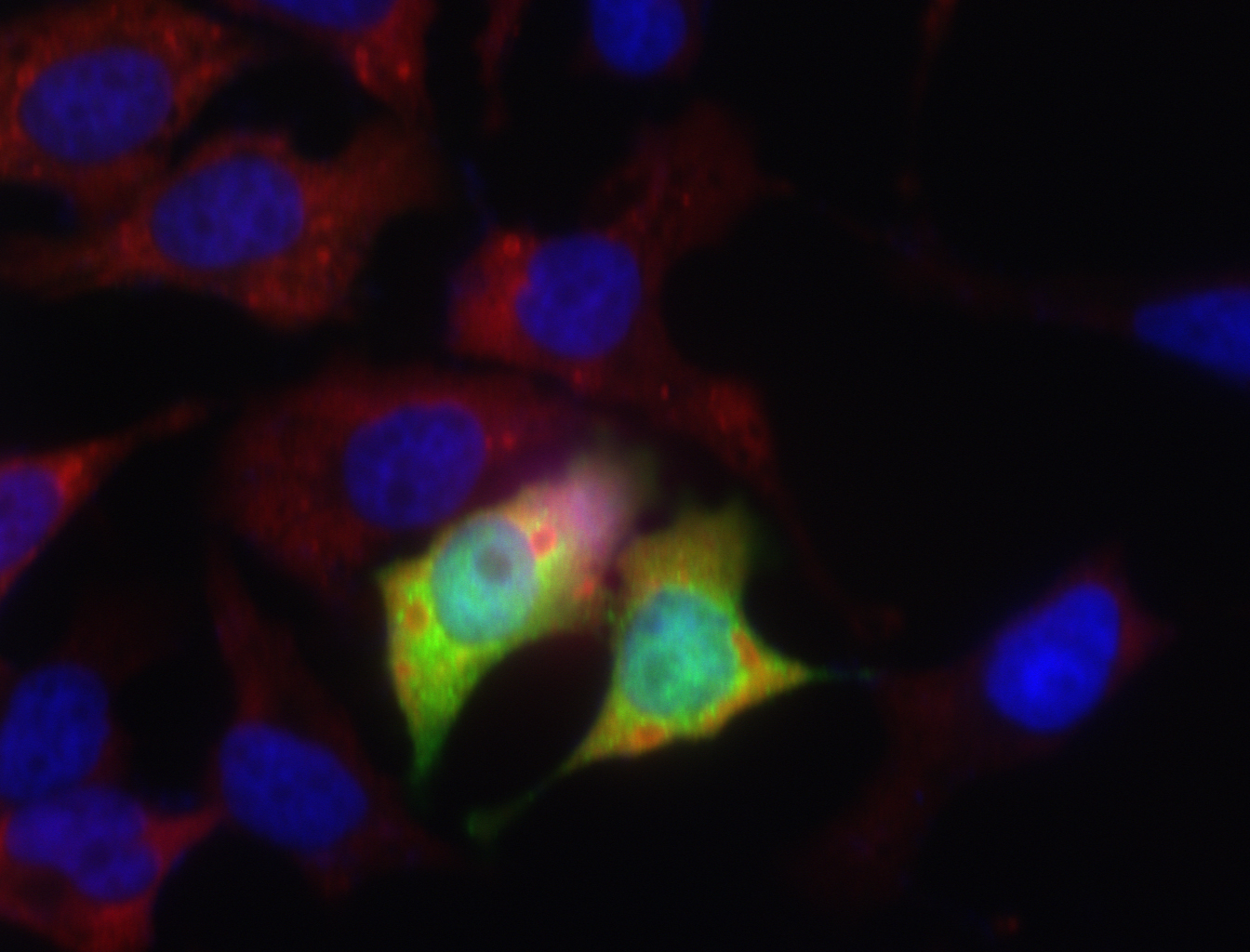

Supplement: Figure 3—source data 1. [file elife-30094-fig3-data1.zip › B_LD401_002&-24Bit.jpg]

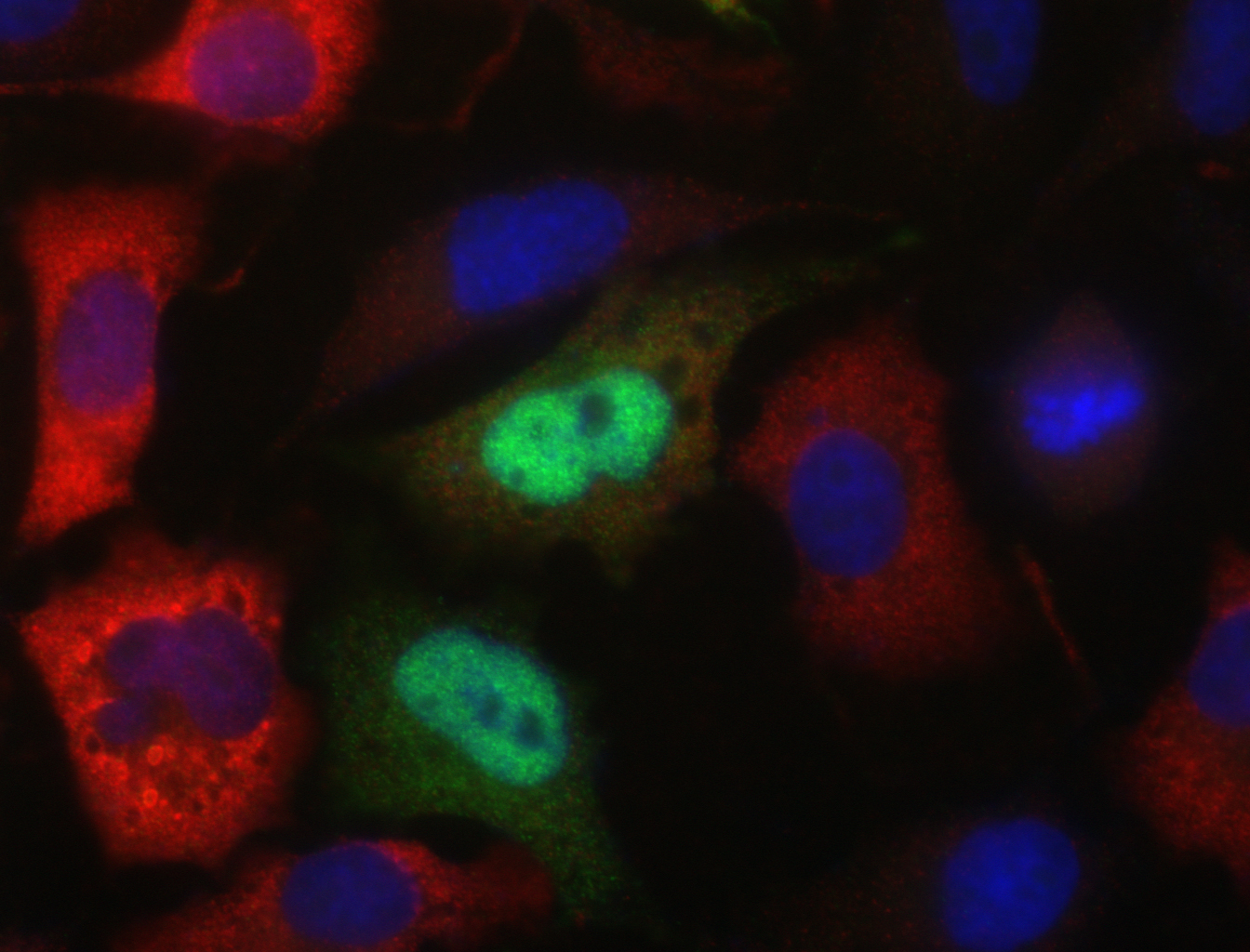

Supplement: Figure 3—source data 1. [file elife-30094-fig3-data1.zip › C_LD401+Fibro_001&-24Bit.jpg]

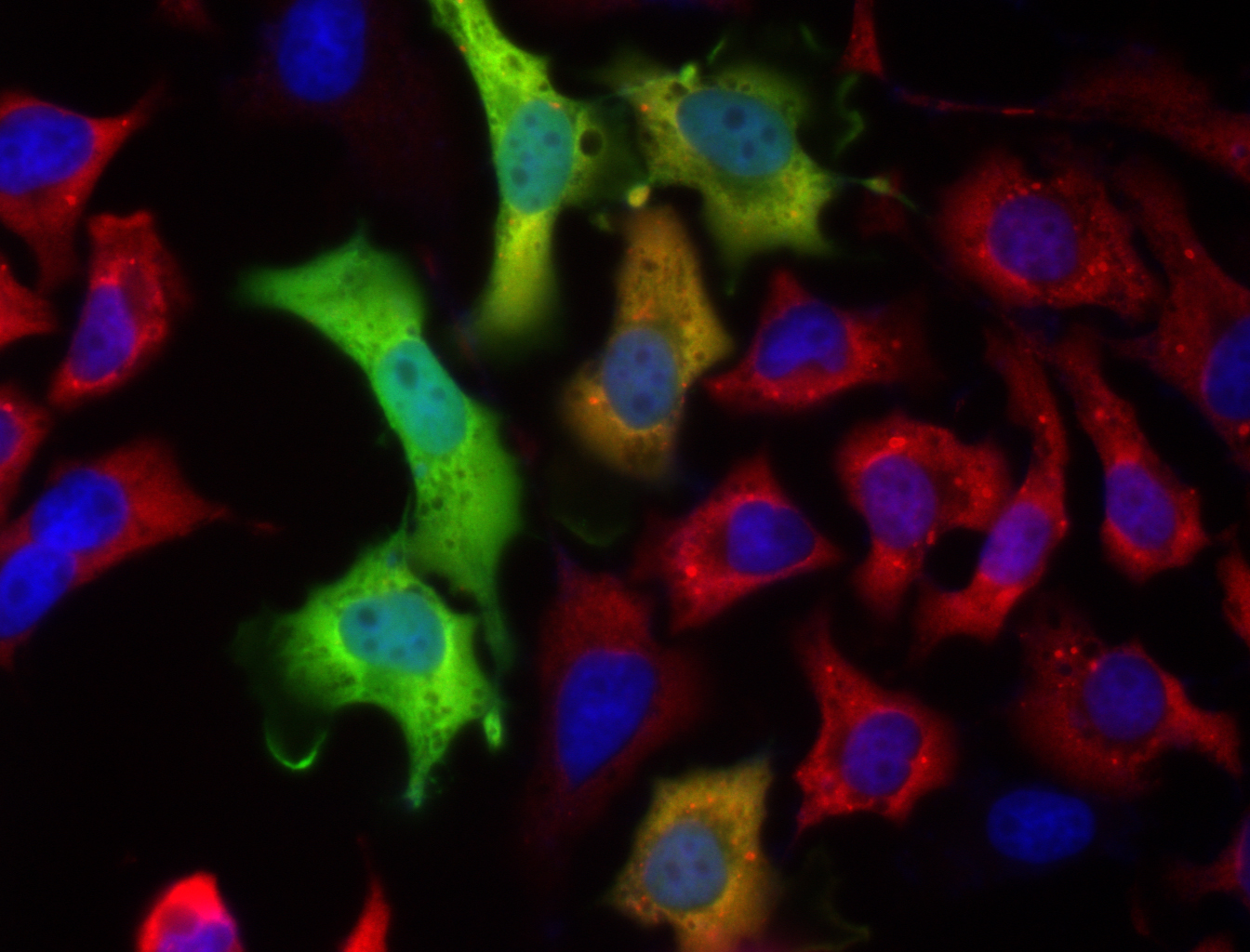

Supplement: Figure 3—source data 1. [file elife-30094-fig3-data1.zip › D_LD401+Fibro_002&-24Bit.jpg]

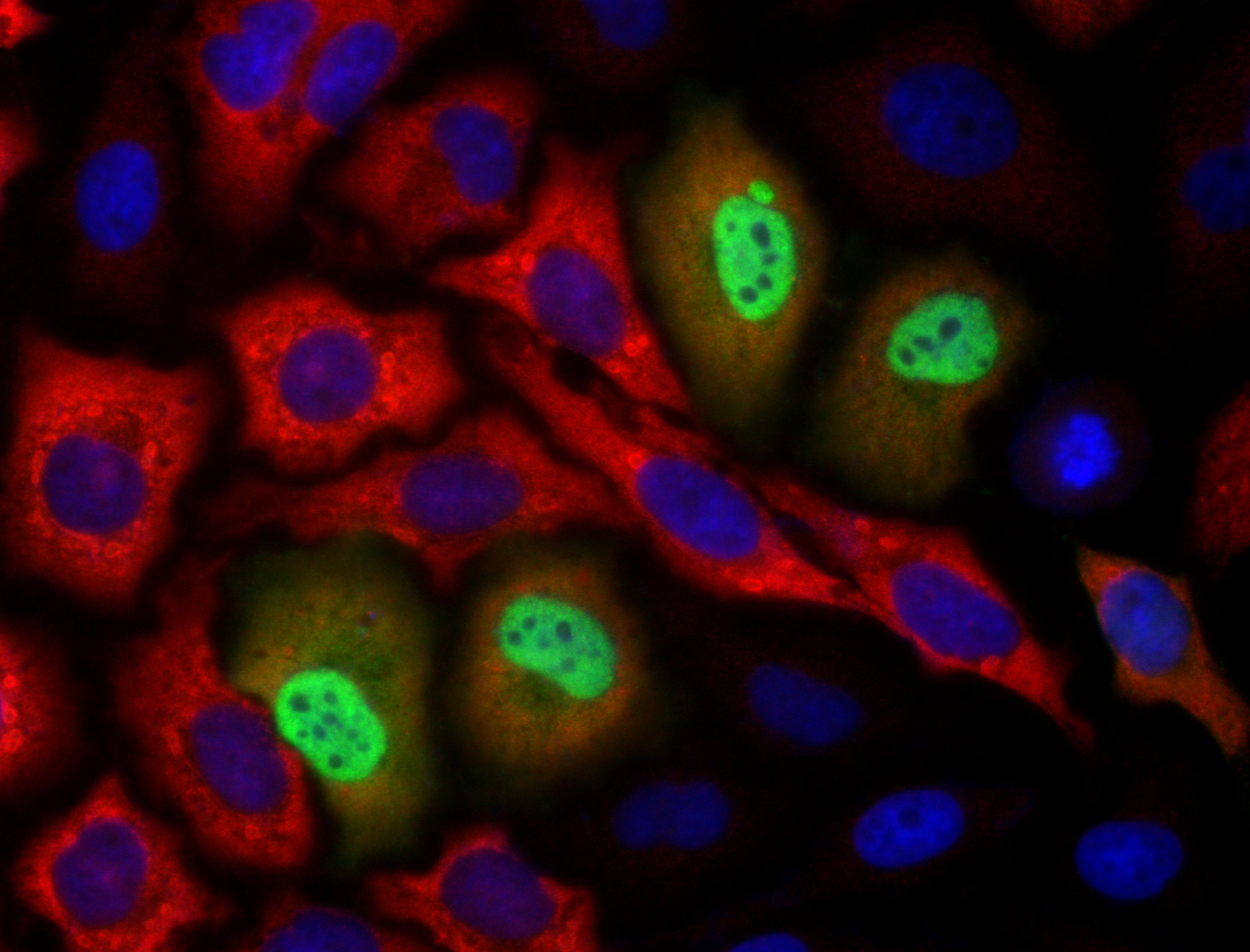

Supplement: Figure 3—source data 1. [file elife-30094-fig3-data1.zip › E_LD401+Fibro_003&-24Bit.jpg]

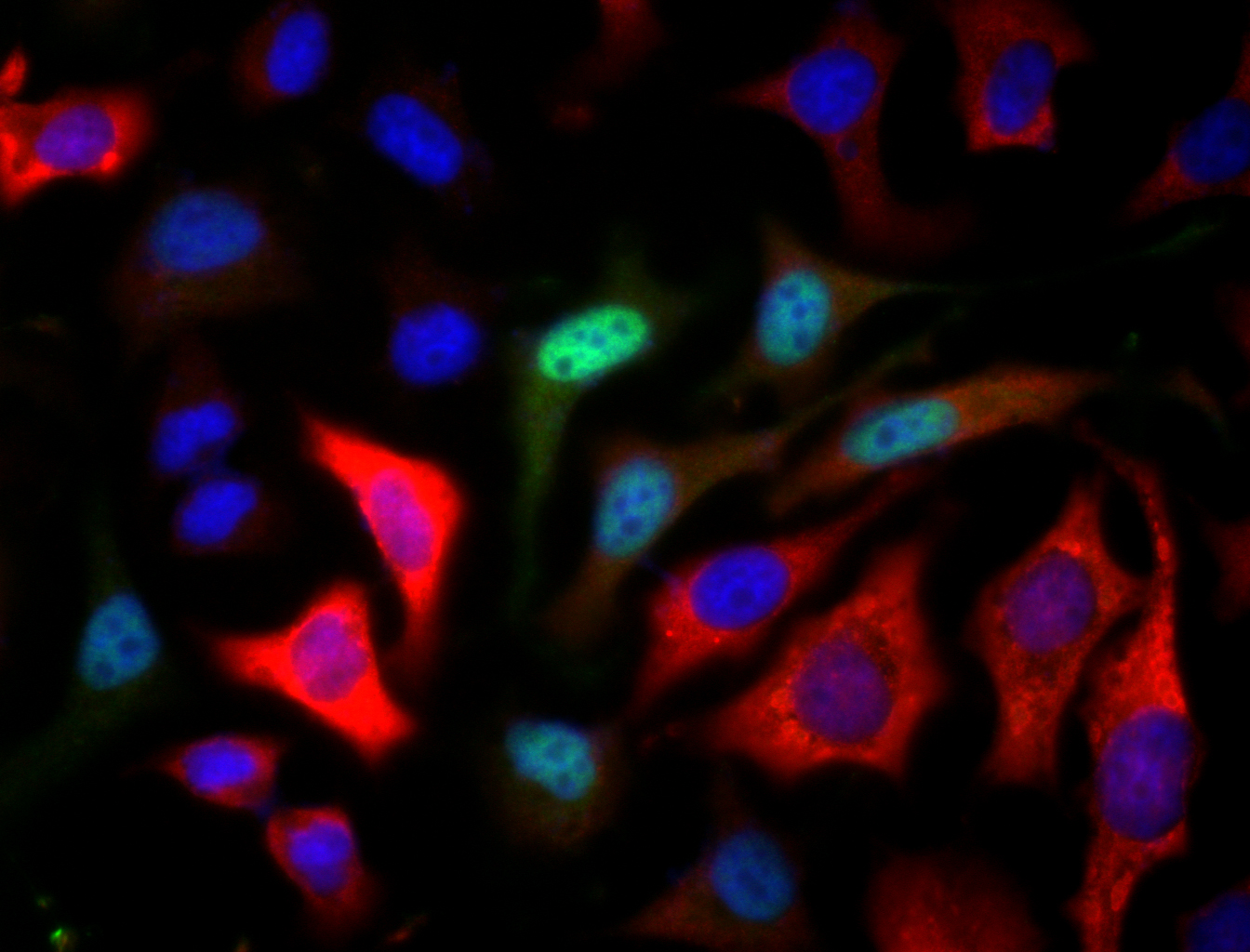

Supplement: Figure 3—source data 1. [file elife-30094-fig3-data1.zip › F_LD401+Fibro_004&-24Bit.jpg]

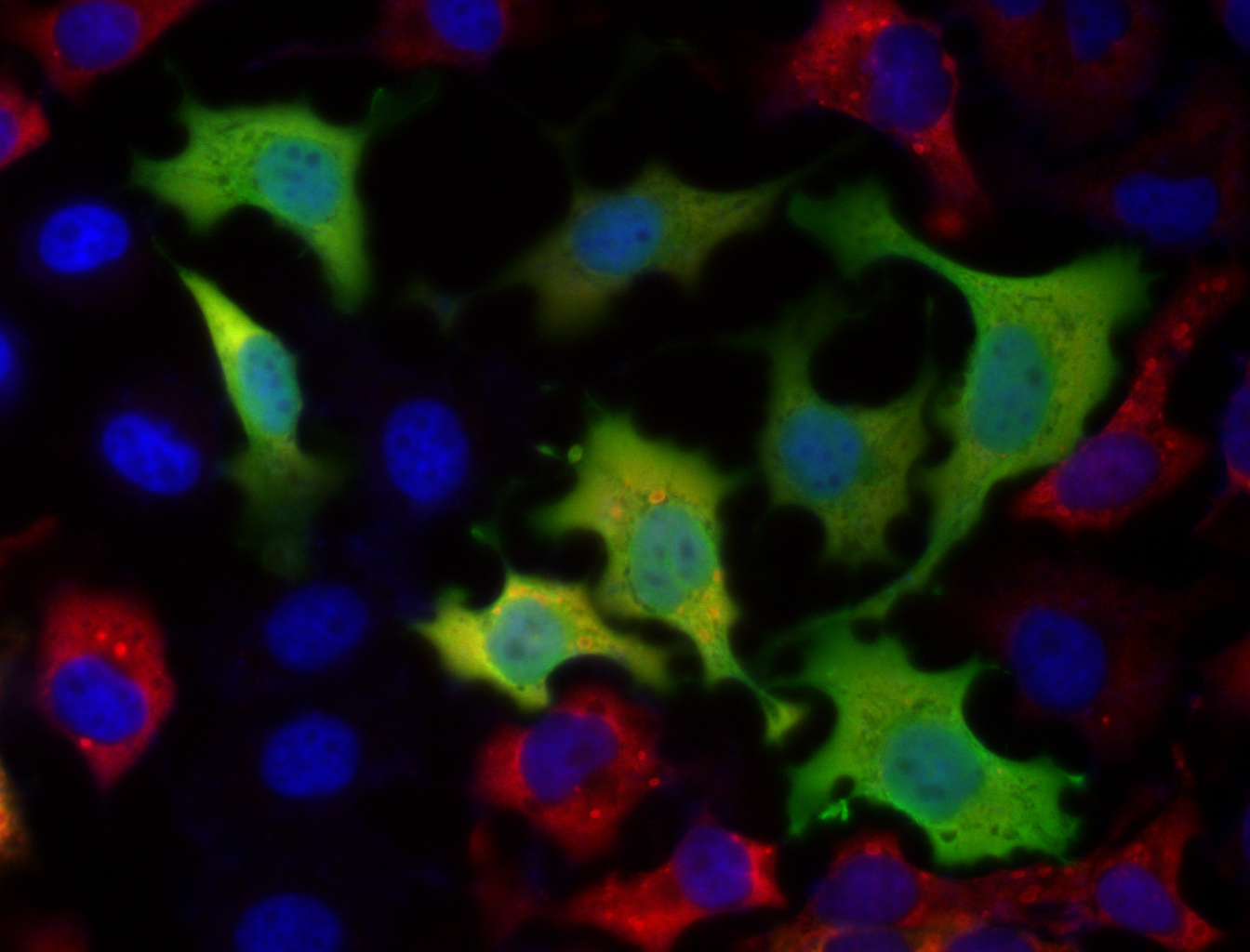

Supplement: Figure 3—source data 1. [file elife-30094-fig3-data1.zip › G_LD401+Fibro_005&-24Bit.jpg]

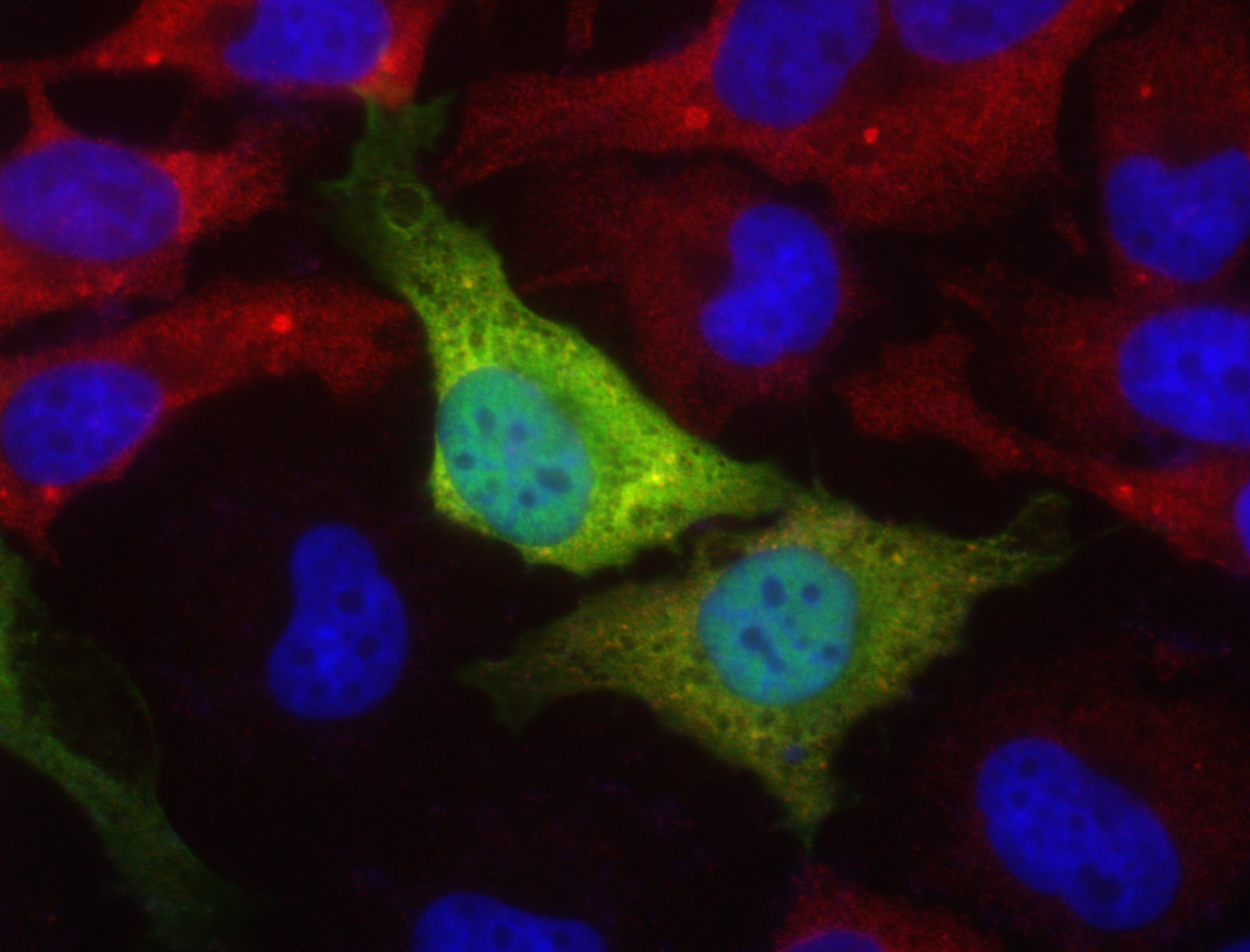

Supplement: Figure 3—source data 1. [file elife-30094-fig3-data1.zip › H_LD401+Fibro_0020&-24Bit.jpg]

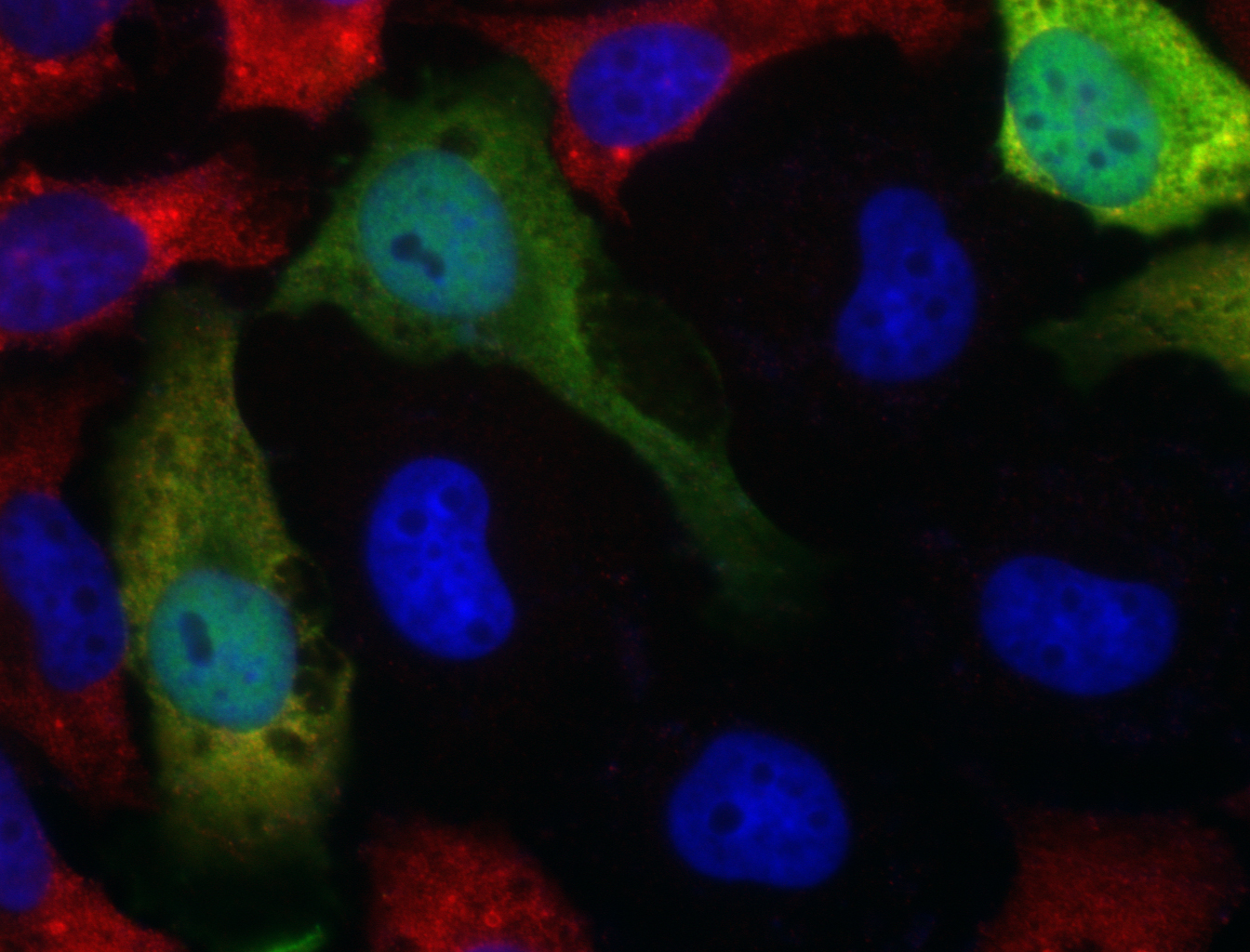

Supplement: Figure 3—source data 1. [file elife-30094-fig3-data1.zip › I_LD401+Fibro_0030&-24Bit.jpg]

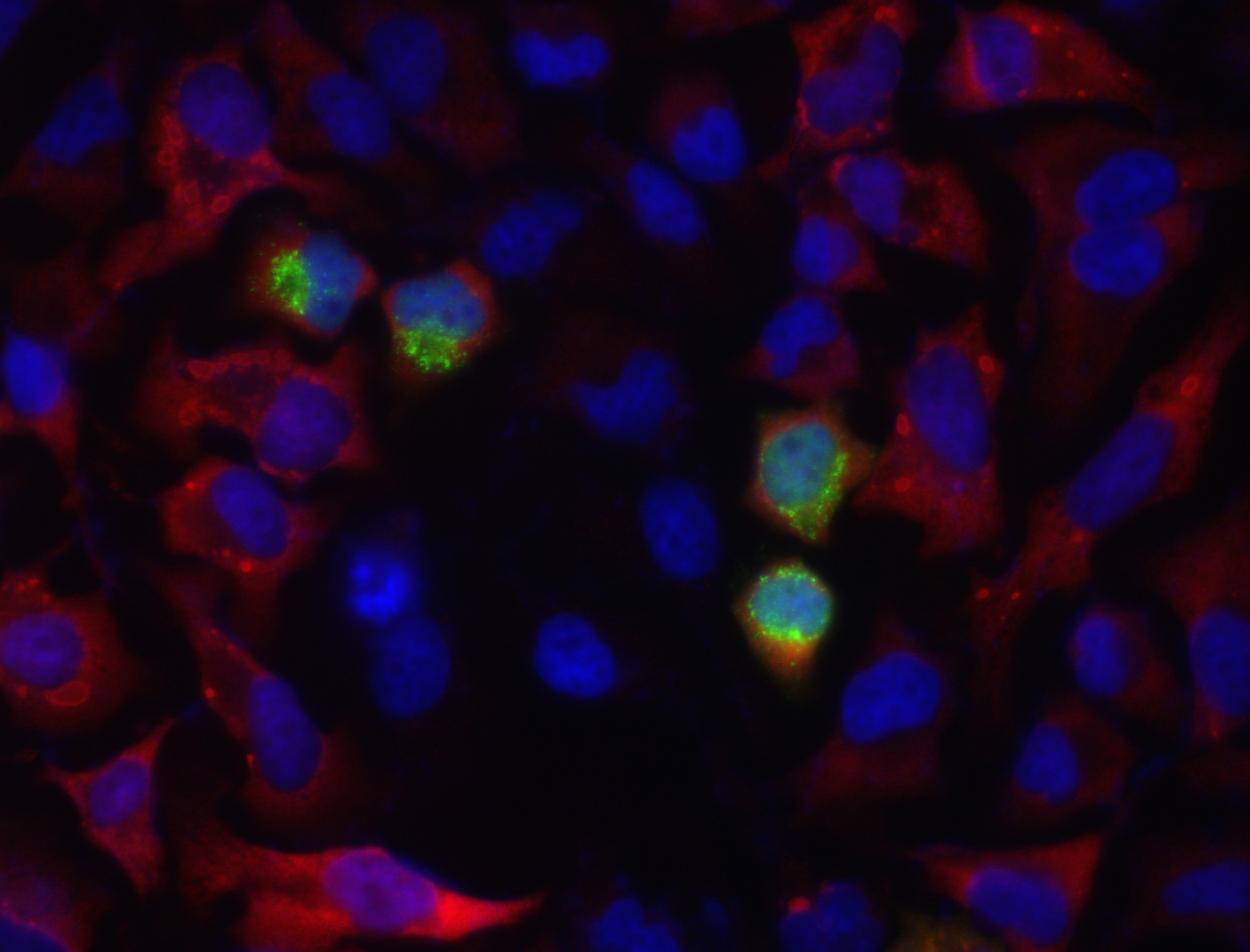

Supplement: Figure 3—source data 1. [file elife-30094-fig3-data1.zip › J_LD401-Fibronectin_002&-24Bit.jpg]

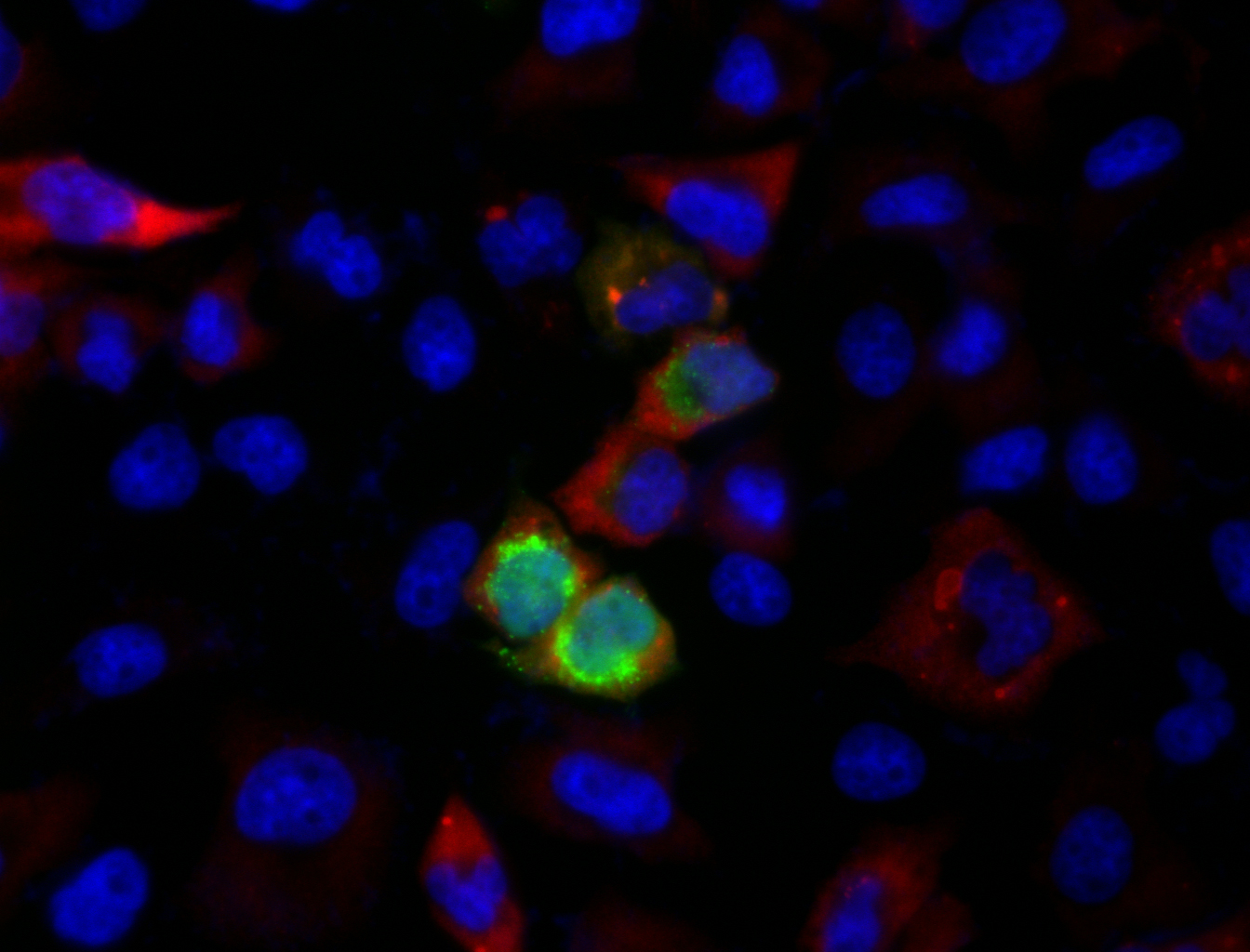

Supplement: Figure 3—source data 1. [file elife-30094-fig3-data1.zip › K_LD401-Fibronectin_003.jpg]
