# Supplementary material for: Dissection of affinity captured LINE-1 macromolecular complexes
Source: eLife. 2018 Jan 8;7:e30094. doi: 10.7554/eLife.30094 (PMC5821459; doi:10.7554/eLife.30094)
Supplement: Transparent reporting form [file elife-30094-transrepform.docx]

***eLife’s* transparent reporting form**

We encourage authors to provide detailed information *within their submission* to facilitate the interpretation and replication of experiments. Authors can upload supporting documentation to indicate the use of appropriate reporting guidelines for health-related research (see [EQUATOR Network](http://www.equator-network.org/%20)), life science research (see the [BioSharing Information Resource](https://biosharing.org/)), or the [ARRIVE guidelines](http://www.plosbiology.org/article/info:doi/10.1371/journal.pbio.1000412) for reporting work involving animal research. Where applicable, authors should refer to any relevant reporting standards documents in this form.

If you have any questions, please consult our Journal Policies and/or contact us: [editorial@elifesciences.org](mailto:editorial@elifesciences.org).

**Sample-size estimation**

- You should state whether an appropriate sample size was computed when the study was being designed
- You should state the statistical method of sample size computation and any required assumptions
- If no explicit power analysis was used, you should describe how you decided what sample (replicate) size (number) to use

Please outline where this information can be found within the submission (e.g., sections or figure legends), or explain why this information doesn’t apply to your submission:

No explicit power analysis was used. Experimental sample size was chosen on the basis of cost, practicality, and availability of materials/reagents for analysis.

**Replicates**

- You should report how often each experiment was performed
- You should include a definition of biological versus technical replication
- The data obtained should be provided and sufficient information should be provided to indicate the number of independent biological and/or technical replicates
- If you encountered any outliers, you should describe how these were handled
- Criteria for exclusion/inclusion of data should be clearly stated
- High-throughput sequence data should be uploaded before submission, with a private link for reviewers provided (these are available from both GEO and ArrayExpress)

Please outline where this information can be found within the submission (e.g., sections or figure legends), or explain why this information doesn’t apply to your submission:

The numbers of replicates are stated in the Methods section and Figure legends. All experiments carried out in this study used cell material produced by large-scale suspension growth. In this scenario, multiple distinctive cultures (biological replicates) of up to 400 ml are combined – producing batches yielding several-to-tens of grams wet cell weight – and the combined materials are cryomilled together. Thus producing material composed of multiple biological replicates. Additionally, when metabolic labeling is used, the light and heavy materials are grown and milled separately, constituting distinctive biological replicates of independently pooled material. Such materials were used for all experiments in this manuscript. Our results as assessed by protein staining, western blotting, and mass spectrometry have been stable for many years (Taylor et al 2013).

Briefly, in Fig. 1, SILAC label-swapped replicates were used. In Fig. 2, duplicates were performed for pLD401 (RNA-seq and LEAP) and pLD567 (RNA-seq), and a single replicate was done for pLD624 (RNA-seq); duplicates were performed for pLD561 (LEAP). In Fig. 4, SILAC labeled samples were combined in triplicate experiments. In Fig. 5, a distinctive single experiment was carried out for each time-point.

For the experiments and preparative methods described, all data recorded have been retained and reported.

**Statistical reporting**

- Statistical analysis methods should be described and justified
- Raw data should be presented in figures whenever informative to do so (typically when N per group is less than 10)
- For each experiment, you should identify the statistical tests used, exact values of N, definitions of center, methods of multiple test correction, and dispersion and precision measures (e.g., mean, median, SD, SEM, confidence intervals; and, for the major substantive results, a measure of effect size (e.g., Pearson's r, Cohen's d)
- Report exact p-values wherever possible alongside the summary statistics and 95% confidence intervals. These should be reported for all key questions and not only when the p-value is less than 0.05.

Please outline where this information can be found within the submission (e.g., sections or figure legends), or explain why this information doesn’t apply to your submission:

Statistical analyses are described in the Methods section, Figure legends, and Appendix. Specifically, for the ORF2p+ nuclei proximity analysis, statistical tests, exact values of N, and exact p-value is stated in Figure legend 3B. More detailed explanation of the statistical analysis of the distances between pairs of ORF2p+ nuclei is described in methods section. For mass spectrometry data analysis, processed data is presented in the figures following normalization procedure described in the methods section and in the Appendix; information about statistical tests and multiple hypothesis correction can be found in the Methods section and P-values were reported throughout the text.

(For large datasets, or papers with a very large number of statistical tests, you may upload a single table file with tests, Ns, etc., with reference to sections in the manuscript.)

**Group allocation**

- Indicate how samples were allocated into experimental groups (in the case of clinical studies, please specify allocation to treatment method); if randomization was used, please also state if restricted randomization was applied
- Indicate if masking was used during group allocation, data collection and/or data analysis

Please outline where this information can be found within the submission (e.g., sections or figure legends), or explain why this information doesn’t apply to your submission:

Samples were allocated on the basis of biochemical treatments – including treatments with enzymes or affinity separation (Fig. 1), on the basis of genetic coding potential (Fig. 2C, Fig. 3A, Fig. 4), or time-scale (Fig. 5).

**Additional data files (“source data”)**

- We encourage you to upload relevant additional data files, such as numerical data that are represented as a graph in a figure, or as a summary table
- Where provided, these should be in the most useful format, and they can be uploaded as “Source data” files linked to a main figure or table
- Include model definition files including the full list of parameters used
- Include code used for data analysis (e.g., R, MatLab)
- Avoid stating that data files are “available upon request”

Please indicate the figures or tables for which source data files have been provided:

Source data was provided for Fig. 3C. Supplementary File 7 includes the ORFeus-Hs sequence from pLD401 used in RNA sequencing alignment. RNA-seq FASTAQ files are available through Gene Expression Omnibus at NCBI: GSE108270. Proteomics raw data are available via ProteomeXchange with identifier PXD008542. R code can be found at: [**https://bitbucket.org/altukhov/line-1/**](https://urldefense.proofpoint.com/v2/url?u=https-3A__bitbucket.org_altukhov_line-2D1_&d=DwMFaQ&c=JeTkUgVztGMmhKYjxsy2rfoWYibK1YmxXez1G3oNStg&r=S_fUBiCcviF6Fdue-UJckB9Wrp0Npp34W_7B69dw-pA&m=cshQqrSwhMsmmCqzPqAq5A5ebYKqLk1yIbdvF8rCLTk&s=htK-v_ysvQija6i4a8Ltd-iK-qtcwsaxBhCTPoJ9xJY&e=)
